# Episodic Ataxia Type 1: Natural History and Effect on Quality of Life

**DOI:** 10.1007/s12311-021-01360-6

**Published:** 2022-06-03

**Authors:** Tracey D. Graves, Robert C. Griggs, Brian N. Bundy, Joanna C. Jen, Robert W. Baloh, Michael G. Hanna, the CINCH Investigators, Joanna J. Jen, Anthony A. Amato, Richard J. Barohn, Angelika F. Hahn, Kimberly Hart, Barbara Herr, Yunxia Wang, Mohammad Salajegheh, Araya Puwanant, Sanjeev Rajakalendran, Yoon-Hee Cha, Jeffrey Krischer, Laura Herbelin, Kristen Roe, Joseph Gomes, Holly Ruhlig, Bonnie Patterson, David Cuthbertson, Rachel Richesson, Jennifer Lloyd

**Affiliations:** 1 MRC Centre for Neuromuscular Disease, UCL Institute of Neurology, Queen Square, London, WC1N 3BG UK; 2 Present Address: Hinchingbrooke Hospital, Northwest Anglia NHS Foundation Trust, Hinchingbrooke Park, Huntingdon, PE29 6NT UK; 3 Department of Neurology, University of Rochester School of Medicine & Dentistry, Rochester, NY 14642 USA; 4 Pediatrics Epidemiology Center, University of South Florida College of Medicine, Tampa, FL 33612 USA; 5 Department of Neurology, UCLA Medical School, Los Angeles, CA 90095-1769 USA; 6 Present Address: Department of Neurology, Icahn School of Medicine at Mount Sinai, 5 East 98th Avenue, New York, NY 10029 USA

**Keywords:** Episodic ataxia type 1, EA1, *KCNA1*, Natural history, SF-36

## Abstract

Episodic ataxia type 1 (EA1) is a rare autosomal potassium channelopathy, due to mutations in *KCNA1*. Patients have childhood onset of intermittent attacks of ataxia, dizziness or imbalance. In order to quantify the natural history of EA1, its effect on quality of life and in preparation for future clinical trials, we set up an international multi-centre study of EA1. We recruited thirty-three participants with EA1: twenty-three completed 1-year follow-up and eighteen completed 2-year follow-up. There was very little accumulation of disability or impairment over the course of the 2 years of the study. The outcome measures of ataxia (SARA and functional rating of ataxia) and the activities of daily living scale were largely stable over time. Self-reported health-related quality of life (SF-36) scores were lower across all domains than controls, in keeping with a chronic condition. Physical subdomain scores appeared to deteriorate over time, which seems to be driven by the female participants in the study. This is an interesting finding and warrants further study. Attacks of EA1 reported by participants in real time via an interactive voice response system showed that symptoms were not stereotyped; however, attack duration and frequency was stable between individuals. This large prospective study is the first ever completed in subjects with EA1. We document the natural history of the disorder over 2 years. These data will enable the development of outcome measures for clinical trials of treatment.

## Introduction

Episodic ataxia type 1 (EA1) is a rare autosomal potassium channelopathy, due to mutations in *KCNA1* [1]. Patients have childhood onset of intermittent attacks of ataxia, dizziness or imbalance [2]. They can also have myokymia, which can manifest as a tremor. As is common with rare diseases, no prospective studies have been performed. Data on treatment, prognosis and natural history are extrapolated from case studies and personal experience. As drug discovery and licensing become stricter, it is likely new compounds will need to be tested on all target groups in small randomised controlled trials. It would be difficult to interpret the effect of such compounds, without more formal information on the natural history of the disease. Therefore, to quantify the natural history of EA1 and its’ effect on quality of life, we set up an international multi-centre study on a large number of patients with EA1. We hope this information will act as a baseline for future clinical trials.

## Materials and methods

### Study design

This was an international, prospective, observational study of the clinical characteristics of EA1. The inclusion criteria have been previously reported [3].

### Outcome measures

We used the Scale for the Assessment and Rating of Ataxia (SARA) as the primary outcome measure to assess disease progression [4]. The evaluator also assigned participants a score for the functional rating stage of ataxia. Health-related quality of life was assessed using the SF-36, a self-administered questionnaire that measures eight quality of life (QoL) domains [5]. Activities of daily living (ADL) assessment tested nine domains, with a maximum score of 36 (0 being no impairment).

### Interactive voice response (IVR) system

At the baseline visit, participants were given orientation to an automated interactive voice response system and were expected to call in every attack for 8 consecutive weeks following recruitment. The flow sheet of questions has been previously reported [3] (https://academic-oup-com/brain/article/137/4/1009/367720#supplementary-data).

### Statistical analysis

Continuous variables that follow a parametric distribution were analysed by Student’s *t* test. *P* values are reported as two-tailed with a cut-off of 0.05 for significance. To measure sex differences in the SF-36 quality-of-life component scores, we fit a random effects model to the physical dimension and, separately, to the mental dimension with the fixed covariants of time and gender. A random effect was assumed for the Y-intercept and based on a likelihood ratio test. The random effect for slope was added for the mental dimension outcome. We included an interaction term between year of observation and gender in an effort to quantify the relationships, not basing them on significance levels due to the small sample size.

### Standard protocol approvals, registrations, and patient consents

The study was approved by the ethics committees of the contributing centres. Written informed consent was obtained from study participants. Oversight for protocol consistency was provided by the data management and coordinating centre, which also performed periodic audits.

## Results

The genotype–phenotype correlation and baseline data from this study have been previously reported [3]. Thirty-three participants with genetically confirmed EA1 were initially enrolled. Twenty-three completed 1-year follow-up and eighteen 2-year follow-up. Reasons for withdrawal were withdrawal of consent (4), loss to follow up after two visits (4), recruited less than 1 year before study completion date (6) or wrong follow-up intervals (1). Although the protocol allowed some leeway for scheduling of annual follow-up visits, these needed to be within 6 weeks of the due date. Several participants missed a year one visit but attended a year two visit and thus could not be analysed to demonstrate year on year changes. The demographics of this group are shown in Table 1. EA1 is thought of as a paroxysmal disease with frequent, brief attacks of cerebellar ataxia interspersed with normality; however, a proportion does go on to develop a persistent cerebellar ataxia (PCA) [3].

**Table 1:** Baseline clinical characteristics of the cohort

Type of EA (n)	Sex n (%)	Age at onset (years)		Age at baseline (years)		Disease duration (years)		Average number of monthly attacks		Prophylactic medication n (%)	SARA total score	
		Mean	SD	Mean	SD	Mean	SD	Mean	SD		Mean	SD
Attacks only 15	Female 9 (60%)	7.67	6.25	35.53	16.43	27.87	15.47	14.2 (range 0–100)	24.95	4 (26.67)	1.6	1.93
Attacks and PCA 3	Female 1 (33%)	12.33	4.99	52	10.19	39.67	14.64	1.67 (range 1–2)	0.47	0	11	2.45
Total 18	Female 10 (67%)	8.44	6.30	38.28	16.73	29.83	15.95	12.11	23.25	4 (22.22)	3.17	4.04

Eighteen participants with genetically confirmed EA1 completed the study. They were divided into those with attacks only and those with both EA1 attacks and persistent cerebellar ataxia (PCA). Those with both EA1 attacks and PCA appeared to be older with longer disease duration and lower attack frequency. However, there was no statistically significant difference (Student’s *t* test) in any of the parameters between the attacks only and PCA groups, although this may be a reflection of the small number of participants in the second group.

### Measures of disability

#### SARA

**Fig. 1:**
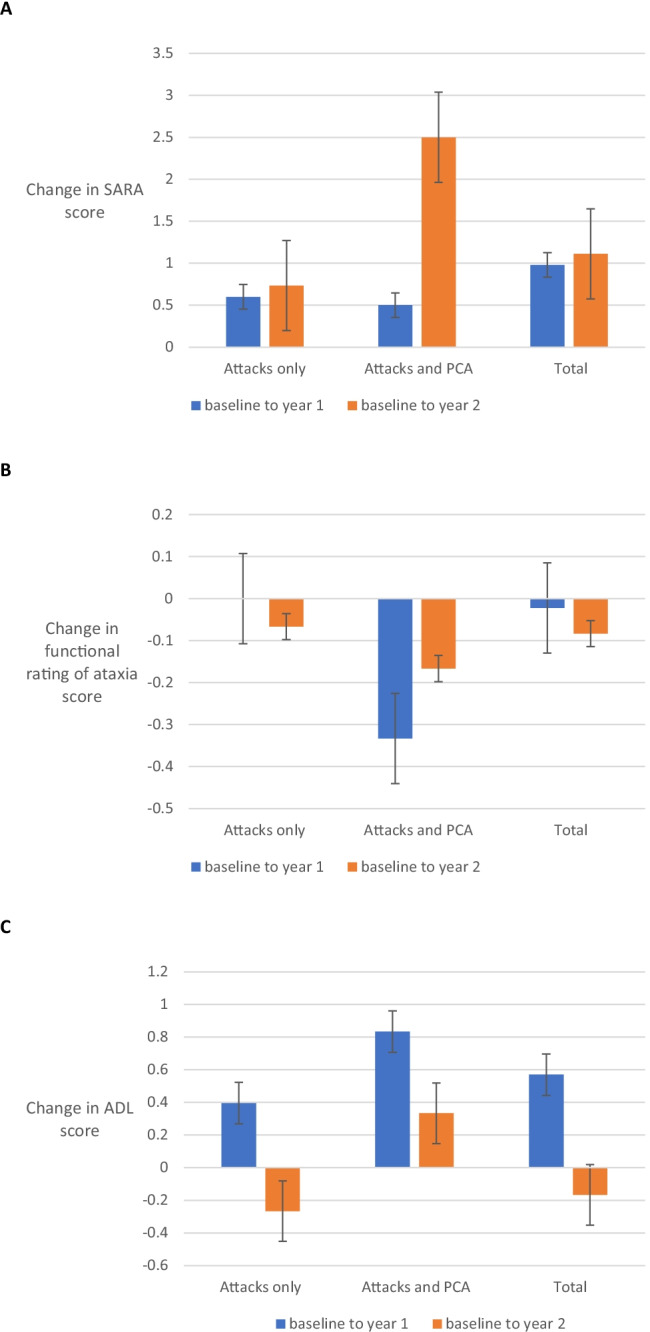
Measures of disability. (**a**) Change in Scale for the Assessment and Rating of Ataxia (SARA) score over the duration of the study. The SARA score ranges from 0 to 40, with 0 indicating no ataxia and 40 the most severe degree of ataxia. Five participants were stable at 0. SARA scores were analysed for each time point for all participants (*n* = 18) and compared between baseline, year 1 and year 2. This analysis was performed for the total cohort as well as both subgroups (EA with attacks alone and EA attacks with persistent cerebellar ataxia (PCA)) to see if one group was influencing the results for the entire cohort. Data shows mean ± SEM. (**b**) Change in functional rating of ataxia over the duration of the study. Evaluator rated classification of the severity of ataxia over three visits (*n* = 18). Functional rating of ataxia ranges from 1 to 6, where 1 represents minimal signs and no disability whilst 6 is total disability. These stages were (1) minimal signs detected by the physician during screening but able to run or jump without loss of balance, representing no disability. (2) Symptoms recognised by the patient but still mild. However, there is an inability to run or jump without losing balance. The patient is physically capable of living an independent life, but daily activities may be somewhat restricted, representing minimal disability. (3) Overt and significant symptoms requiring regular or periodic holding on to wall or furniture or use of a stick for stability and walking, representing moderate disability. (4) Requiring a frame, crutches or two sticks to walk, being able to perform several activities of daily living, representing moderate disability. (5) Confined but can navigate a wheelchair, performing some activities of daily living that do not require standing or walking, representing severe disability. (6) Confined to wheelchair or bed with total dependency for all activities of daily living, representing total disability. Where the evaluator felt that the status was about the middle between two stages, an increment of 0.5 could be used. Functional rating of ataxia scores were analysed for each time point for all participants (*n* = 18) and compared between baseline, year 1 and year 2. This analysis was performed for the total cohort as well as both subgroups (EA with attacks alone and EA attacks with persistent cerebellar ataxia (PCA)) to see if one group was influencing the results for the entire cohort. Data shows mean ± SEM. (**c**) Change in activities of daily living (ADL) score over the duration of the study. An activities of daily living (ADL) assessment covered the ability to wash, dress, swallow, breathe, walk, sit, toilet and whether there were any falls, with a maximum score of 36 (0 being no impairment). This was a self-reported assessment. Total activities of daily living scores were analysed for each time point for all participants (*n* = 18) and compared between baseline, year 1 and year 2. Most participants (*n* = 9) had a stable score of 0. This analysis was performed for the total cohort as well as both subgroups (EA with attacks alone and EA attacks with persistent cerebellar ataxia (PCA)) to see if one group was influencing the results for the entire cohort. Data shows mean ± SEM

The mean score at baseline was 3.17 ± 0.98 (mean ± SE), at year one 1.73 ± 0.88 and at year two 2.44 ± 0.99 (*n* = 18). The SARA score ranges from 0 to 40, (0 indicating no ataxia and 40 severe ataxia), suggesting EA1 is mild when compared with other forms of ataxia. Fifteen (83.33%) participants remained stable or improved, and the remaining three (16.67%) deteriorated. A proportion of EA1 patients (21%) develop persistent cerebellar ataxia [3]. Participants with persistent cerebellar ataxia (*n* = 3) had higher SARA scores (mean at baseline, 11) which improved with time (year one, 12, year two, 10.33). In those with attacks only (*n* = 15), the mean SARA score at baseline was 1.6 ± 0.51, which also improved with time (year 1 1.0 ± 0.52, year 2 0.9 ± 0.52) (Fig. 1a). The most likely explanation for this was a current or recent EA1 attack at baseline, not present at other visits. Of those with attacks only, two participants’ scores deteriorated (mean change 1), six improved (range 1–5, mean change 2.17 ± 0.65) and the remaining seven remained stable. In those with persistent cerebellar ataxia, gait, speech, heel-shin test and the finger-nose test were the highest scoring elements on the SARA, suggesting both appendicular and midline ataxia. In contrast, no cerebellar eye signs were elicited and sitting balance was normal.

#### Functional rating of ataxia

The functional rating of ataxia ranges from 1 to 6, where 1 represents minimal signs and no disability, whilst 6 is total disability. In our cohort, scores were largely stable over time. Thirteen participants (72.22%) showed no change and four (22.22%) deteriorated while one (5.56%) improved. Eighty percent of participants with attacks only (*n* = 15) remained stable at 1.0 over the study period, and the remaining three are increasing from 1.0 to 1.5. In those with persistent cerebellar ataxia (*n* = 3), one remained stable at 1.0, one improved and one deteriorated. Thus, there was very little change when looking at the entire cohort (Fig. 1b).

#### Activities of daily living (ADLs)

This is a subjective measure, with participants answering questions on aspects of activities of daily living with a 5-point scale for each question. The scale ranges from 0 to 36. Most participants (50%) scored 0 at baseline and at subsequent visits (*n* = 9), suggesting no impairment in ADLs. Three participants showed deterioration in their scores (range 1–4, mean change 3.33 ± 1.2), and five showed an improvement (range 1–3, mean change 1.6 ± 0.4), whilst the remaining participant remained stable on a score of 1. These gains were made in the falling category, perhaps suggesting they were suffering EA1 attacks at baseline which was not the case in subsequent visits. In those participants whose scores deteriorated, this was over a range of domains. Thus, there was very little change when looking at the entire cohort over time (Fig. 1c).

The two participants with the highest scores suffer from persistent cerebellar ataxia. It would be expected for them to have more impairment than those with brief attacks. At baseline the mean ADL score for those with EA1 attacks only was 0.47 ± 0.19, by year two it was 0.73 ± 0.07 (*n* = 15). If those with persistent cerebellar ataxia are included, these means rise to 0.94 ± 0.42 at baseline and 1.11 ± 0.42 at completion (*n* = 18). Given scores can go up to 36, the effect of EA1 on ADLs is mild, with or without persistent cerebellar ataxia.

Two participants without persistent cerebellar ataxia ADL scores increased at year two from 0 at baseline to 4 and 5, respectively, both were female. This was not correlated with an increase in their SARA or functional rating of ataxia scores. One participant had SF-36 data available, which showed a decline in physical health scores, but this was no more dramatic than that seen in other females.

### Health-related quality of life (QoL)

Although twenty-three participants were seen for follow-up visits at year one and eighteen at year two, we only have SF-36 responses from eighteen participants for year one and sixteen for year two. This is because some questionnaires were lost in the post (5 in year one, 2 in year two). In addition, three participants were seen at the wrong follow-up intervals, so had data at baseline and year two and this was not captured at year one. Given the small number of participants, we decided to analyse all available data.

**Fig. 2:**
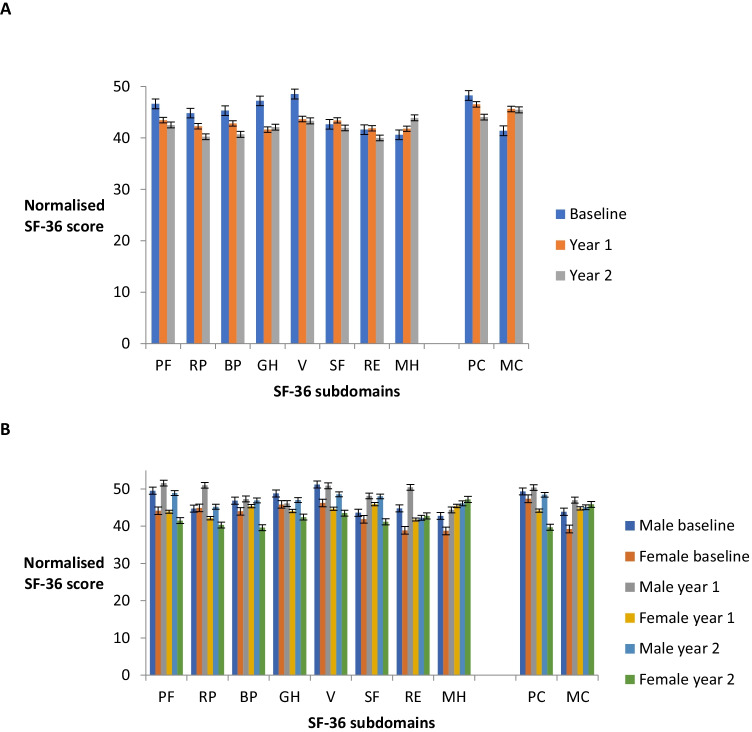
Quality of life. (**a**) Change in SF-36 health-related quality of life over the duration of the study. Self-reported health-related quality of life measure (SF-36) subdomain normalised scores from baseline to year two (*n* = 16). Error bars are SEM. (**b**) Change in the SF-36 health-related quality of life measure over the duration of the study according to gender. Comparison between self-reported health-related quality of life measure (SF-36) subdomain normalised scores of male and female participants from baseline to year two (male n = 8, female n = 8). Error bars are SEM. PF, physical functioning; RP, role physical; BP, bodily pain; GH, general health; V, vitality; SF, social functioning; RE, role emotional; MH, mental health; PC, physical component score; and MC, mental component score. The component scores are aggregates of the 8 subdomains, divided into physical (physical functioning, role physical, bodily pain, general health) and mental categories (vitality, social functioning, role emotional, mental health)

The results are grouped into eight subdomain scores which contribute to two overall scores for physical and mental health. The scores are normalised any below 50 are below the average for a reference population [5]. Mean scores across all domains were lower in participants with EA1 than the general population, in keeping with a chronic condition (Fig. 2a). Physical health subdomain scores and vitality deteriorated over time. Mental health domains remained stable or improved. Given the small numbers, none of these reached statistical significance.

**Fig. 3:**
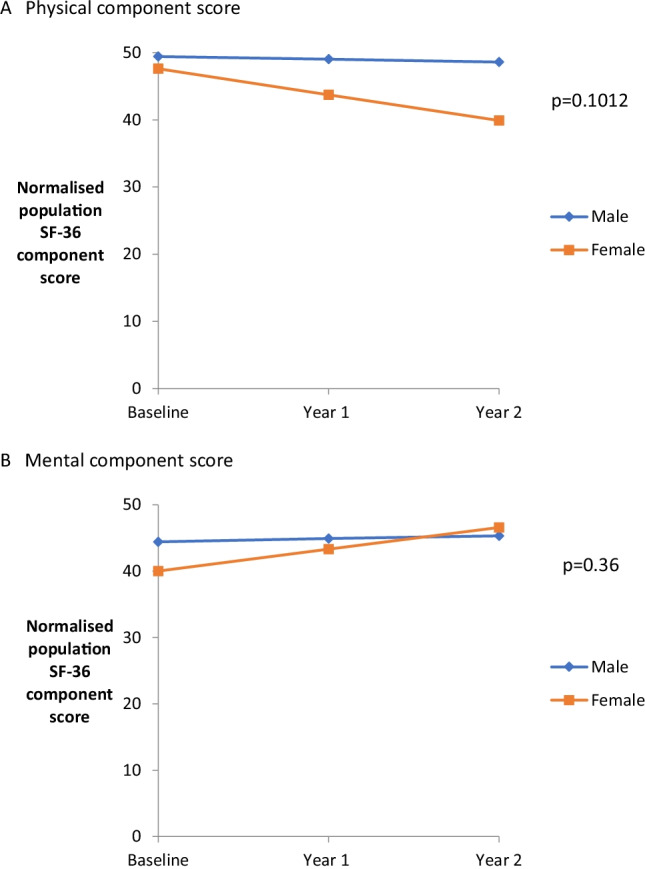
Quality of life according to gender. Comparison between the sexes for the SF-36 health-related quality of life physical and mental component scores using a random effects model. Self-reported health-related quality of life measure (SF-36) physical and mental component scores were analysed according to gender from baseline to year two (male *n* = 8, female *n* = 8). The population physical and mental component normalised score means were derived using a random effects model and compared. The component scores are aggregates of the 8 subdomains, divided into physical (physical functioning, role physical, bodily pain, general health) and mental categories (vitality, social functioning, role emotional, mental health). (**a**) Physical component score. Comparison between means on the self-reported health-related quality of life measure (SF-36) physical component scores according to gender. (**b**) Mental component score. Comparison between means on the self-reported health-related quality of life measure (SF-36) mental component scores according to gender

When these means were analysed by gender (male *n* = 8, female *n* = 8), the scores were lower in females (20 of 24 data comparisons) (mean difference 5.07, minimum difference 1.75, maximum difference 8.82) (Fig. 2b). This is particularly prominent in the physical rather than mental health subdomains. When all scores were plotted individually, physical function deteriorated year on year for females, whereas the mental function seemed to improve. Scores for males were stable in both domains. Using a random effects model on the population means, this trend based on gender suggested an association which did not reach statistical significance (*p* > 0.10), possibly due to the small sample numbers. The results are displayed in the graph based on this model, which shows the population means (Fig. 3a and b). Therefore, it appears to be the deterioration in the perceived physical health of the female participants which is driving the trend in the whole group.

### Patient reported symptoms—interactive voice response (IVR) system

**Fig. 4:**
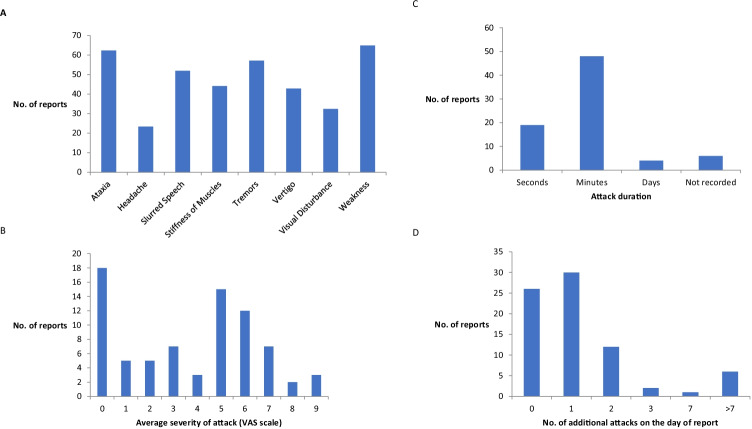
Self-reported symptoms captured by the interactive voice response (IVR) system. Data on attacks was obtained from 6 individuals and 77 attacks over the course of the study. Participants were asked a series of yes/no questions on an automated system to record the nature of their attacks. (**a**) Symptoms during attacks. Participants were asked what symptoms they had during the current attack (yes/no). (**b**) Attack severity. Participants were asked to rate the severity of the effect of the attack, where 0 is not severe and 9 is very severe (visual analogue scale). (**c**) Attack duration. Participants were asked to record the duration of the attack. (**d**) Number of additional EA1 attacks suffered on the day of reporting. In order to increase compliance, participants were not required to call with every attack on a given day but described the symptoms for one attack and then recorded the number of additional attacks suffered on that day

Participants were asked to capture data on attacks in the 8 weeks following each visit, to reduce their workload and hence increase compliance. This depended upon participants (a) having attacks and (b) remembering to call with the data. Any participant who did not respond were contacted weekly to remind them of this part of the study, to maximise compliance. One participant had attacks at baseline and year 1 follow-up, two reported attacks at baseline and year two follow-up and three reported attacks at all three time points. In total, these six individuals made seventy-seven episode reports via the IVR during the course of the study. Attack features were variable but largely similar within individuals. Due to the small numbers completing the IVR over 3 years, it is difficult to make any comment on changes to symptoms over time. Contrary to expectations, weakness was the most common symptom reported (64.9% of attacks), rather than ataxia (62.3% of attacks) (Fig. 4a). Attacks were classified as having a mild to moderate (88.3%) effect on daily function. The most frequent severity score was 0 the next being 5, suggesting a bimodal distribution (Fig. 4b). Attack duration was usually seconds to minutes (87%) (Fig. 4c). Attack frequency was largely stable within individuals over time (Fig. 4d).

## Discussion

This large study of EA1 provides the first prospective data on the natural history of the disorder. In general, both objective and subjective outcome measures showed stable scores over 2 years. During the study, little disability accumulated, which is not surprising, given the paroxysmal nature of the condition. The objective outcome measures of cumulative disability by the ataxia rating scale (SARA) and functional rating of ataxia were largely stable over time. SARA scores in our cohort were generally low, in keeping with the episodic nature of the condition, despite an average disease duration of 27.6 years. This contrasts with other inherited ataxias, which have permanent progressive symptoms. In the common autosomal dominant spinocerebellar ataxias (SCAs), the average SARA at baseline was higher than our cohort at 15 (with average disease duration of 10 years). Deterioration of the SARA was seen after 1 year of follow up, although it varied according to type, showing a more aggressive decline in SCA1 (2.11 points/year), when compared to SCA6 (0.8 points/year) [6]. In Friedreich’s ataxia (FRDA), the commonest recessive ataxia, the mean SARA at baseline was even higher, at 22 (average disease duration of 17 years) with an average annual decline in the SARA of 1.36 points/year [7]. In our cohort, the functional rating of ataxia proved to be an ineffective tool at demonstrating change, as thirteen participants showed no change, four had deteriorated another one improved over the duration of the study. This reflects the lack of cumulative disability, as seen in the SARA scores, and could probably have been predicted due to the intermittent nature of the condition.

EA1 appears to cause minimal impairment as shown by the ADL scale, which was largely stable over time. Despite no apparent effect on ADLs, EA1 significantly affects the QoL in this cohort. SF-36 scores were reduced across all domains compared to controls, in keeping with a chronic condition. Interestingly, they were similar in most domains to those previously reported in FRDA, where patients are significantly more disabled [8]. However, as patients with EA1 are not severely affected by physical disability, scores for physical functioning were much lower in the FRDA cohort (22 c.f. 42 at year 2 of this study). In our cohort, scores in physical not mental domains seemed to deteriorate over time. This was not statistically significant, given the small numbers and the effect was still evident when those with persistent ataxia were removed from the analysis. When analysed separately with a random effects model, this trend could be seen to be driven by the female participants in the study. This result is interesting and warrants further investigation. Previous studies in gender difference in QoL in patients with neurological disease have shown variable results. In Chinese stroke patients, scores in six out of the eight SF-36 domains were lower in female compared to male participants, despite being well-matched for age, stroke size and co-morbidities. Female sex was the major factor affecting recovery [9]. In Huntington’s disease, motor symptoms seemed to have a greater effect on QoL for females, whereas cognitive symptoms drove changes in QoL in male participants [10]. In the SCAs, male subjects scored lower than their female counterparts in four domains (three out of four of the physical domains), and this only reached statistical significance for general health, despite having eighty participants [11]. Therefore, it is difficult to make any conclusions for our cohort, with a relatively small sample size. A meta-analysis of studies in MS studies found no gender difference in QoL [12], a condition which affects more women than men, suggesting these affects may be disease-specific.

Attacks of EA1 reported by participants in real time showed attacks were usually mild in severity with a mild to moderate effect on daily functioning. Symptoms were not stereotyped, but attack duration and frequency were stable between individuals. Therefore, these would be the most useful outcome measure for future clinical trials. Contrary to expectations, weakness was the most common symptom reported, rather than ataxia, which was a close second. The IVR system was useful at collecting real time data and could be valuable for future drug studies. We had tried to minimise the number of calls participants would have to place, to make the study less onerous. This resulted in less data being captured than we had hoped, as patients could go weeks to months without any attacks, especially if they were on preventative medications. Therefore, it would have to be used for the entire duration of any future clinical trials, to ascertain as much data as possible. Compliance with the use of the IVR system could be increased by daily reminder text messages or emails, which would be computer-generated with no greater workload on investigators. For studies in rare diseases, there will always be small numbers of participants, so it is imperative to reduce the drop-out rate where possible. We contacted participants in advance to schedule annual appointments and were flexible up to 12 weeks around their due date. They were offered travel expenses, but no other financial incentives, and were contacted after their visit if no IVR responses were logged. Novel measures may be required to increase retention rates in future studies [13]. In a similar study performed by the CINCH group into the non-dystrophic myotonias, maintaining participation was also an issue. The original cohort reported ninety-three participants [14]. The publication of the IVR findings only report seventy-six participants [15]. Even within this partial data set, compliance was only 63.4% for once weekly IVR over an 8-week period in this natural history study [15]. Participation in a drug trial did little to increase compliance. A similar IVR protocol was the primary outcome measure in a trial of mexiletine in non-dystrophic myotonia, with 74.3% compliance for daily IVR calls collected over 4 weeks [16]. A Cochrane review suggested the best mechanism for increasing compliance was the offer of a small monetary incentive (up to £20) for completion of postal or electronic questionnaires [17]. This may need to be considered in future for data collection tools which on previous evidence would appear to be more onerous on participants than the trial designers anticipated, i.e., daily telephone calls for IVR. Performing a pilot study or involving patients in trial design could also be helpful.

For future studies, it would be prudent to document recent attacks around the time of study visits, changes in treatment or additional diagnoses, to try to reduce confounding effects.

In conclusion, we have characterised the natural history of EA1 over 2 years and documented its effect on QoL. Real-time acquisition of information about EA1 attacks has revealed the symptoms are not stereotyped, although their duration and frequency are mostly stable within individuals. Our research shows rare diseases can be studied in the same way as common conditions, although this requires disease registries and a concerted international effort. We provide important data for the planning of future clinical trials in the paroxysmal conditions, which will be necessary to find and licence new treatments for these orphan diseases.

## Appendix

The Consortium of Clinical Investigation on Neurologic Channelopathies (CINCH):

Steering Committee: Principal Investigator: Robert W. Baloh, MD (UCLA, CA). Site Principal Investigators: Joanna J. Jen MD, PhD (UCLA, CA), Anthony A. Amato, MD (Boston, MA), Richard J. Barohn, MD (Kansas City, KS), Robert C. Griggs, MD (Rochester, NY), Michael G. Hanna, MD (London, UK), Angelika F. Hahn, MD, (London, ON). Chief Statistician: Brian N. Bundy, PhD (Tampa, FL). CINCH Lead Research Coordinator: Kimberly Hart (Rochester, NY) and CINCH Program Manager: Barbara Herr, MS (Rochester, NY). Investigators/CINCH Trainees: Yunxia Wang, MD (Kansas City, KS), Mohammad Salajegheh, MD (Boston, MA), Araya Puwanant, MD (Rochester, NY), Tracey D. Graves, PhD, FRCP (London, UK), Sanjeev Rajakalendran, MRCP, PhD (London, UK), Yoon-Hee Cha MD, (UCLA, CA). Data Management and Coordinating Centre Principal Investigator: Jeffrey Krischer, PhD (Tampa, FL). Clinical Evaluators: Laura Herbelin, BS (Kansas City, KS). Clinical Coordinators: Laura Herbelin, BS (Kansas City, KS), Kimberly Hart (Rochester, NY), Barbara Herr, MS (Rochester, NY), Kristen Roe (Boston, MA). Participating Members of the Data Management and Coordinating Centre: Joseph Gomes (Tampa, FL), Holly Ruhlig, MS, ARNP, CCRC (Tampa, FL), Bonnie Patterson, BA CCRP (Tampa, FL), David Cuthbertson, MS (Tampa, FL), Rachel Richesson, PhD, MPH (Tampa, FL) and Jennifer Lloyd (Tampa, FL).

## Abbreviations

ADLs: Activities of daily living
EA1: Episodic ataxia type 1
IVR: Interactive voice response
QoL: Quality of life
PCA: Persistent cerebellar ataxia
SARA: Scale for the Assessment and Rating of Ataxia
SF-36: Short form 36
