# Correction to: Episodic Ataxia Type 1: Natural History and Effect on Quality of Life

**DOI:** 10.1007/s12311-022-01450-z

**Published:** 2022-07-29

**Authors:** Tracey D. Graves, Robert C. Griggs, Brian N. Bundy, Joanna C. Jen, Robert W. Baloh, Michael G. Hanna, the CINCH Investigators

**Affiliations:** 1 MRC Centre for Neuromuscular Disease, UCL Institute of Neurology, Queen Square, London, WC1N 3BG UK; 2 Hinchingbrooke Hospital, Northwest Anglia NHS Foundation Trust, Hinchingbrooke Park, Huntingdon, PE29 6NT UK; 3 Department of Neurology, University of Rochester School of Medicine & Dentistry, Rochester, NY 14642 USA; 4 Pediatrics Epidemiology Center, University of South Florida College of Medicine, Tampa, FL 33612 USA; 5 Department of Neurology, UCLA Medical School, Los Angeles, CA 90095-1769 USA; 6 Department of Neurology, Icahn School of Medicine at Mount Sinai, 5 East 98th Avenue, New York, NY 10029 USA

**Correction to: The Cerebellum**

**https://doi.org/10.1007/s12311-021-01360-6**

The original version of this article unfortunately contained some errors.

Some editorial comments were inserted into figure legend 3 by mistake and the intended corrections in legend subpanels were not made.

The original article has been corrected.

